# Worth your sweat: wearable microfluidic flow rate sensors for meaningful sweat analytics

**DOI:** 10.1039/d4lc00927d

**Published:** 2025-01-22

**Authors:** R. F. R. Ursem, A. Steijlen, M. Parrilla, J. Bastemeijer, A. Bossche, K. De Wael

**Affiliations:** a Electronic Instrumentation, Department of Microelectronics, Delft University of Technology Mekelweg 4 2628 CD Delft The Netherlands a.s.m.steijlen@tudelft.nl; b Antwerp Engineering, Photoelectrochemistry and Sensing (A-PECS), University of Antwerp Groenenborgerlaan 171 2020 Antwerp Belgium marc.parrillapons@uantwerpen.be karolien.dewael@uantwerpen.be; c NANOlab Center of Excellence, University of Antwerp Groenenborgerlaan 171 2010 Antwerp Belgium

## Abstract

Wearable microfluidic sweat sensors could play a major role in the future of monitoring health and wellbeing. Sweat contains biomarkers to monitor health and hydration status, and it can provide information on drug intake, making it an interesting non-invasive alternative to blood. However, sweat is not created in excess, and this requires smart sweat collection strategies to handle small volumes. Microfluidic solutions are commonly employed which use capillary action or evaporation to drive flow. In current literature about sweat analytics, the emphasis lies predominantly on developing the sensors for measuring the composition of sweat. Yet, solely measuring sweat composition does not suffice, because the composition varies due to inter- and intra-individual differences in sweat rate. The measurement of sweat rate is thus crucial for enabling a reliable interpretation and standardisation of this data. Recently, more wearable sweat sensors, also integrating a means of measuring flow, have been developed. This manuscript reviews state-of-the-art sweat collection strategies and flow rate measuring techniques. Generally, flow rate measurements are performed by impedimetric or capacitive methods. However, these techniques can be impaired due to limited lifetime and signal interference from changing ionic contents in sweat. Discrete measurement techniques, such as impedance measurements of an advancing fluid front with interdigitated electrodes, calorimetric and colorimetric techniques can be very reliable, because they selectively measure flow. However, one should take the available size, intended application and compatibility with other sensors into account. Overall, accurate flow rate sensors integrated in reliable microfluidic sweat sensor platforms will enable the standardisation of sweat measurements to unlock the potential of sweat analytics in advancing physiological research, personalized diagnostics and treatment of diseases.

## Abstract 2

This review focuses on recent microfluidic flow rate sensing methods for reliable sweat sensing, covering physiological relevance and providing technical insights.

## Graphical abstract

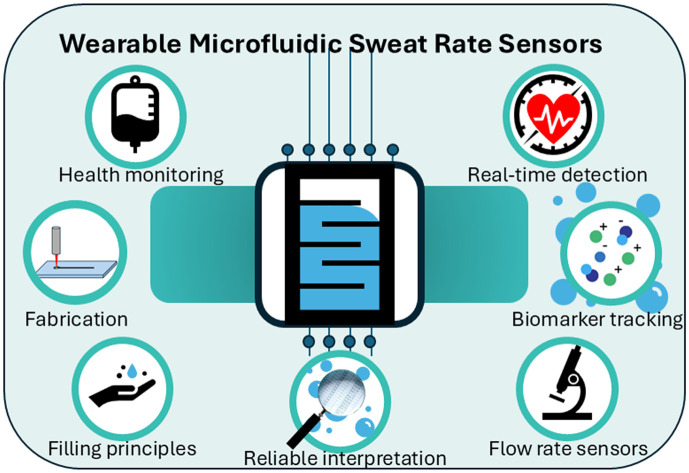

## 1 Introduction

Wearable sweat sensors can provide a non-invasive, continuous method of monitoring health.^1,2^ This is because sweat can be easily collected from the skin for drug monitoring and physiological analysis.^3–7^ The non-invasive nature of sweat sensing facilitates greater patient comfort, as it reduces the need for invasive blood analysis. Using sweat for analysis can also enable decentralised monitoring of patients in the clinic and at home.

While non-invasive health monitoring has attractive prospects, wearable sweat sensors have to overcome several multidisciplinary challenges in their development. These challenges include mechanical, chemical and electronic engineering principles. For example, the devices have to ensure wearer comfort and excellent contact for collection, often by matching the skin's elastic properties. Besides, wearable sweat sensors require microfluidics to handle small volumes due to the low perspiration rates. All the while, the sensors must offer great sensitivity to the target analyte, which is usually only present in low concentrations, and they must be robust in the presence of interferents.^2^ Various types of sensors have been used for the analysis of the composition of sweat. Among them, electrochemical sensors and colorimetric sensors have been widely used due to their miniaturisation-ability and simplicity.^2,8–11^ With the use of electrochemical sensors, sweat can be swiftly analysed after collection using techniques such as potentiometry and voltammetry. However, electrochemical measurements can be difficult to interpret due to the susceptibility to irregular flow rates, interferents, pH, or temperature, which might alter the analytical output of the sensors. On the other side, colorimetric sensors might be susceptible to light conditions and subjective interpretation (when not employing standardisation conditions).^12^

Relative to other biofluids for analytical purposes, sweat itself actually has a suitable matrix. This is due to the absence of excessive amounts of lipids and proteins. Instead, eccrine sweat predominantly contains water, electrolytes and metabolites in smaller concentrations.^2,13,14^ For information on wearable sweat sensors for the analysis of sweat composition, the reader is referred to these reviews.^2,9–11,14–16^ Sweat, however, does not have a constant composition. Rather, its contents vary according to the sweat rate. The sweat rate varies per person based on age, diet, sex, ethnicity and environment.^13^ The natural sweating response can vary within a person depending on the state of physical activity, psychogenic stress and body location.^17^ Even when sweating is artificially stimulated, the sudomotor response varies between individuals. This is important to consider since sweat rate can influence the concentration of the analyte in sweat, which is often neglected.^13,18,19^ Normalising for sweat rate can account for this variability. Sweat rate sensors thus play a critical role in developing a platform for monitoring biomarkers or (therapeutic) drugs in sweat.

**Fig. 1:**
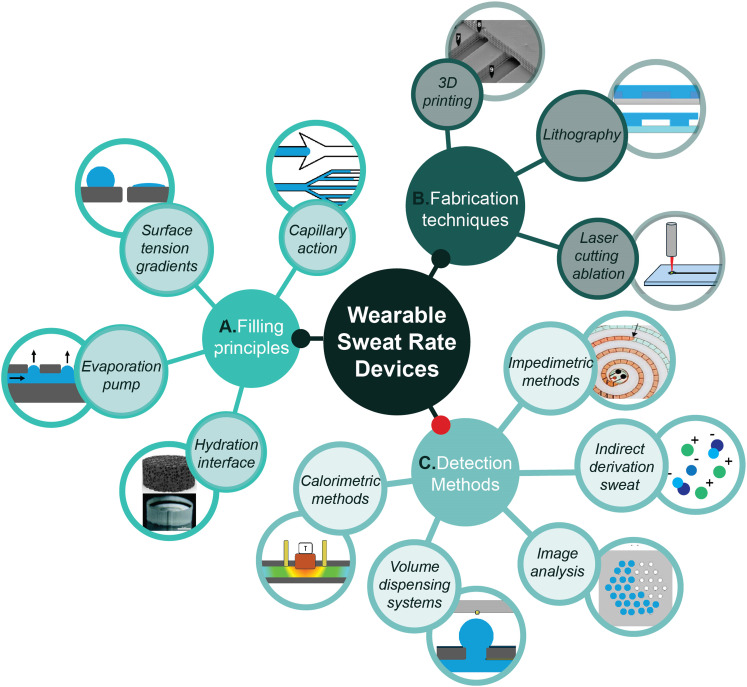
Schematic overview of the A) filling principles, B) fabrication techniques and C) detection methods involved in the development of wearable microfluidic sweat rate devices. Reproduced with permission.^20–23^ Hydration interface upper icon: Copyright 2024, *Biomicrofluidics*. Hydration interface lower icon: Copyright 2017, *Lab Chip*. 3D printing icon: Copyright 2017, *Anal. Chem.* Impedimetric methods: Copyright 2022, *ACS Sens.*

Recently, wearable sweat sensors integrating a means of measuring flow have been developed. To identify the most promising sweat rate sensor principles, it is valuable to investigate the currently used flow rate sensors and evaluate them on performance and efficiency. This review will focus on sweat rate sensing, by first providing insights into the relevance of measuring sweat rate, followed by common principles to determine microfluidic flow in a sweat patch. Subsequently, various fabrication methods will be discussed and the penultimate section will elaborate on the existing flow rate sensors used in sweat sensing applications. Finally, the future prospects of wearable microfluidic sweat rate sensors are discussed. Fig. 1 depicts the three major topics of the review, spanning from the filling principles and fabrication methods of microfluidics devices towards state-of-the-art detection methods for wearable flow rate sensors. The review focuses entirely on the relevance and state of the art of wearable microfluidic sweat rate sensors in the last years due to the dramatic increment of the scientific and societal relevance of this topic. This review aims to serve as a guideline for further development of flow rate sensors by outlining various working principles to measure a flow on the order of nL min^−1^ in wearable configurations. Importantly, it identifies the advantages and pitfalls of certain techniques and suggests future steps in sweat rate sensor development.

## 2 Relevance of sweat as analytical matrix

Wearable sweat sensors are gaining relevance as a tool for continuous health and fitness monitoring by assessing electrolyte levels and hydration status. Moreover, they can measure drug levels more frequently due to their non-invasive nature, which can be used to tailor the medicine dosage. This can be done for therapeutic drug monitoring purposes such as in chemotherapy and antibiotics treatment.^24–27^ Interestingly, the sweat matrix has also been used to detect opioids and MDMA.^28,29^ A major advantage of sweat analysis is that it is non-invasive, as opposed to blood analysis.

### 2.1. Influence of sweat rate on sweat composition

Where blood analysis can provide consistency in results for many biomarkers across a wide population, sweat is more dynamic, because the contents of sweat (99% water) and the sweat secretion rate are influenced by age, fitness, diet, sex, ethnicity and body site.^13^ This renders it rather difficult to identify proper healthy ranges of analytes. Moreover, sweat rate partially determines the composition of sweat. The secretion mechanisms of naturally occurring sodium (Na^+^), chloride (Cl^−^), and potassium (K^+^) have been well studied, while the exact pathways for other analytes remain less understood.^13,30^ A higher sweat rate can lead to analyte concentration because the resorption rate of Na^+^ and Cl^−^ cannot compete with the sweat production rate. Conversely, the impact of sweat rate on the potassium concentration is minimal.^31^ On the other hand, higher sweat rates can lead to analyte dilution because the analyte secretion is slower than the water secretion at higher sweat rates.^10,30^ Because of these effects, it is particularly important to include sweat rate measurements to perform meaningful sweat analysis.

At rest, sweat production per gland is limited to nanoliters per minute, making it challenging to collect sufficient volumes for accurate analysis. Thus, sweat production can be stimulated naturally through exercise or heat, or artificially by iontophoresis. The differences in sweat rate persist ranging from 0.12 μL cm^−2^ min^−1^ up to around 1 μL cm^−2^ min^−1^.^17^ Critically, there seems to be a significant difference between sweat rates of male and female subjects, which can be attributed to differences in body mass and metabolic heat production.^30,32^ Yet, this underlines that only considering sweat composition could affect the interpretation of results for a major part of the population. Healthcare would thus greatly benefit from wearable sweat sensors and continuous sweat analysis without a potential bias towards sex.

This means also incorporating sweat rate sensors in the biochemical analysis,^25^ as it can account for the variability to a great extent.^19^ Harshman *et al.* (2021)^19^ investigated the sweat composition and rates of eleven men who were subjected to two exercise routines for sweat collection on the forearms. Interestingly, they noted a variance between the left and right forearm sweat rate, but most importantly: after a sweat ion conductivity analysis and global metabolomic analysis they found that, respectively, 77.8% and 72.7% of the variation in their measurements could be corrected with sweat rate normalisation, reiterating the importance of accurate localised sweat rate determination for generating reliable results.^19^

### 2.2. Diagnostic opportunities of sweat rate sensors

For cystic fibrosis, sweat analysis is clinically adopted as a diagnostic tool,^13^ but therapeutic drug monitoring in sweat can also be promising.^6,10^ For brevity, a few novel applications are mentioned below, all requiring a sweat rate sensor for reliable results.

People that could benefit from sweat analysis, are patients with obesity who are receiving antibiotic treatment. Due to a higher fat mass, antibiotic absorption is impaired. This requires frequent monitoring of the drug levels in blood.^33,34^ Doing this in sweat would provide a convenient, non-invasive and laboratory-independent way of monitoring the drug level. It might also convey more information for better decision-making processes. Antibiotic tissue penetration can vary within patients and sweat antibiotic levels are downstream from the site of infection. This means that the antibiotics have passed the tissue before entering the sweat. Thus, the concentration of the antibiotics in sweat may resemble the antibiotic levels at the site of infection more closely than in blood, which can be deemed upstream.^35^ Still, standardisation of sweat measurements must be considered for the potential use in drug monitoring.^19,36^

**Fig. 2:**
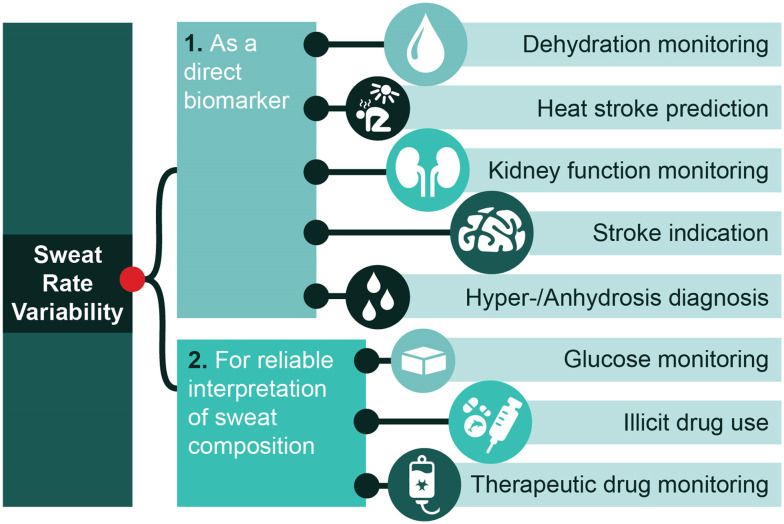
Examples and opportunities for sweat rate sensor applications. Sweat rate has potential to be used as a direct biomarker for various conditions (point 1) or for reliable interpretation of sweat composition (point 2) by accounting for sweat rate effects.

In combination with measuring ionic concentration, sweat rate can also be used as a direct biomarker to assess a person's hydration status.^6,37^ This is not only convenient in sports for the determination of the dehydration threshold and the optimal hydration point for maximising physical output (Fig. 2). There are also many benefits in healthcare. For example, knowing one's fluid balance can be critical in heart- and kidney failure.^6^ In dialysis patients, knowing the hydration is critical but hard to monitor. Some patients become anuric or have very low urine production due to impaired kidney function, which results in a low frequency of analysis.^38^ Other methods, such as imaging the inferior vena cava or using bio-impedance spectroscopy can also be adopted.^39,40^ As sweating is continuous, though significantly reduced in some groups of dialysis patients,^41^ sweat sensors could provide a new, simple method of determining fluid status in dialysis patients in the future. In addition, in the case of certain pathologies, sweat rate may also be altered and thus be used as an indicator, such as hyperhidrosis, anhidrosis, kidney failure or impaired kidney function and strokes.^6,18^ For other opportunities where a sweat rate sensor is necessary for sweat composition analysis, the reader is referred to these reviews.^2,10^

Research in both the physiological and technological measurement of sweat is necessary to provide reliable diagnostics by using wearable sweat sensors. To this end, a wearable microfluidic sweat sensor should include both a chemical sensor and a flow rate sensor, as composition and flow are intertwined. While microfluidic flow sensors are commonly used in other fields of study, sweat has added complexity due to the low excretion volumes and low and irregular flow rates. Resting thermoregulatory sweat is produced at 0.02 nL per min per gland at its minimum.^10^ In activity, this can increase significantly to rates up to 15 nL per min per gland depending on site.^17,18^ While a higher production of sweat reduces the time of analysis, physical exercise cannot serve as a viable means for the medical monitoring of bed bound patients or those with limited mobility. To induce more sweat production and thus flow, artificial sweat stimulants such as pilocarpine, acetylcholine or carbachol *via* a iontophoresis patch can be used.^3,32,42^

## 3 Microfluidic sweat rate sensors

This section proceeds with illustrating various fillings principles to drive flow. To further provide insights, the fabrication possibilities for wearable microfluidic patches are discussed. Finally, the current detection methods are discussed for detecting sweat rate.

### 3.1. Filling principles

**Fig. 3:**
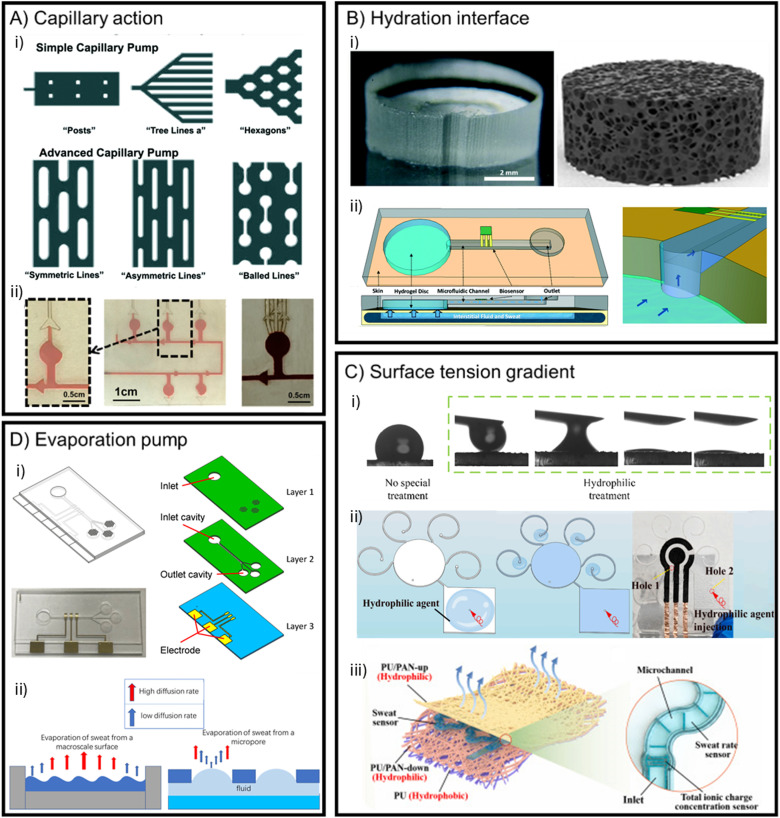
Filling principles in microfluidic sweat patches. A) Techniques to promote and direct flow, showcasing i) simple and advanced capillary pumps to facilitate quicker filling reproduced with permission.^43^ Copyright 2018, *Lab Chip*. ii) The implementation of capillary burst valves can be seen with red colouring reproduced with permission.^44^ Copyright 2023, *Biosensors*. B) Hydration interfaces to draw sweat and minimise dead volume. i) Respectively, a hydrogel and porous graphene to illustrate the common mechanisms, osmotic pressure and capillary porosity for hydration interfaces, reproduced from Shay *et al.*, 2017^23^ and Fu *et al.*, 2024,^22^ respectively. Copyright 2017, *Lab Chip* and Copyright 2024, *Biomicrofluidics*. ii) Implementation of a hydrogel at the inlet of a microfluidic patch, reproduced with permission.^23^ Copyright 2017, *Lab Chip*. C) Illustrations of surface tension (gradients). i) Difference between a hydrophobic surface and a hydrophilically treated surface, resulting in a low surface tension, reproduced with permission.^22^ Copyright 2024, *Biomicrofluidics*. ii) Loading of the hydrophilic agent to create a gradient in surface tension. The agent is injected in the outlet, reproduced with permission.^4^ Copyright 2024, *Talanta*. iii) A Janus textile with a hydrophobic and hydrophilic side, containing a PDMS microfluidic channel for sweat (rate) analysis, reproduced with permission.^45^ Copyright 2023, *Biosensors and Bioelectronics*. D) Examples of an evaporation pump mechanism. i) Implementation of micropore arrays at the end of a microfluidic patch for sweat analysis, reproduced with permission.^46^ Copyright 2019, *Micromachines*. ii) Illustration of mechanism behind evaporation pumps, where diffusion rates are elevated at the edge, opposite to the macroscale, adapted with permission.^47,48^ Copyright 2011, *Lab Chip* and Copyright 2020, *Nanotechnology and Precision Engineering*.

This paragraph first explains the relevance of microfluidics in sweat sensing and the mechanism behind capillary action (3.1.1), which is exploited to drive filling in microfluidic sweat patches. This is further expanded with methods to further facilitate efficient sweat collection. Understanding these principles provides a qualitative basis for designing a flow rate sensor (Fig. 3). Most importantly, the principles explained are convenient for a better comprehension of the next section about detection methods (*i.e.* impedimetric methods or volume dispensing systems). The principles will include: I) hydration interface (3.1.2), II) surface tension gradients (3.1.3), and III) evaporation pumps (3.1.4).

Sweat samples can be collected by using absorbent materials, scraping methods and arm gloves/bags.^2,5,8,42,49^ These require separate analysis and manual handling of sweat.^42^ In additions, these techniques face limitations in contaminating or altering the sweat sample, such as the arm bag method, where moisture accumulation can accelerate desquamation, potentially leading to high mineral concentrations.^8^ Another way to collect sweat is through microfluidic patches on the skin, directly transporting sweat to a sensing area without handling steps.^2^ Microfluidics enhances sample collection by minimising sample leakage, evaporation and contamination, whilst also improving the accuracy of the measurements. Microfluidic channels limit evaporation and allow for an accurate, fixed volume of sweat to be processed, which improves the reliability of the analysis.^9^ Using a flow also limits contamination through constant renewal of the fluid. An additional challenge with microflow sensing, however, is analyte diffusion decreasing the resolution over time. For sodium and chloride ions, the diffusive flux is expected to be on the same order of magnitude as the convective flux in a 100 μm × 100 μm channel, using Fick's law^50^ and fluid flow approximations. For a larger channel, the diffusion will be more dominant. Smaller channels, resulting in faster filling rates, reduce the diffusion effects. Diffusion in low flow rate environments majorly influence the analysis of sweat components.

While microfluidic channels are typically etched in glass or silica, this is not practical for a wearable microfluidic sweat sensor. In patch form, a wearable sweat sensor needs to conform to the flexibility of the body for user comfort and a robust attachment to the skin. The material of the patch contacting the skin should therefore match the properties of the skin.^9^ In addition, it should be able to harness microfluidic channels for fluid transport and storage. Traditionally, microfluidic flow can be controlled with the use of valves and complex external control modules and pumps. For our application, these would not be compatible. This means that for portable sweat detection, novel driving forces must be investigated.^47^ An overview of these driving forces can be found in Fig. 3 and Table 1, showing the filling principle, the fabrication techniques, sweat induction type and the target of analysis. Flow rate is often not targeted when investigating composition.

**Table 1:** Overview of filling principles for recent microfluidic sweat sensors. ns = not specified, PL = photolithography, IP = iontophoresis, LIG = laser-induced graphene, R2R = roll-to-roll, SAM = hydroxyl-terminated self-assembled monolayer, ECB = electronic circuit board, AG-GLY = agarose-glycerol, PU = polyurethane

Device	Filling principle	Target	Body location	Sweat induction	Fabrication	Volume	Ref.
Resettable discrete flow rate sensor	Capillary action + suction	Flow rate	Forearm	Exercise	Laser cutting, PDMS, SAM, PET	10 μL	20
Capillary sweat patch	Capillary action	Na^+^, K^+^, glucose, flow rate	Various	Exercise, IP	R2R, laser cutting, PET, PDMS	24.3 μL	51
Capillary sweat patch	Capillary action	Glucose, lactate	Lower back	Exercise	PL, molding, PDMS	8.24 μL	52
Capillary sweat patch	Capillary action	Na^+^, Cl^−^, K^+^, pH	Forearm	Exercise	PL, molding, PDMS	14 μL	53
Hydrogels for sweat induction	Capillary action, hydrogel IP	Glucose	Forearm	Integrated IP	Etching, molding, PDMS	3.2 μL	54
Low cost microfluidic sweat patch	Capillary action	Na^+^, Cl^−^	Back	Exercise	Laser cutting, PET–PU coating	10 × 70 μL	55
Microfluidic evaporation pump	Capillary action, evaporation	Na^+^, K^+^, glucose, lactate	Upper arm	Exercise	PL, molding, laser cutting, PDMS with 0.5% Triton X-100	16 μL	56
Compartment filling microfluidic patch	Capillary bursting valves	None	Various	Exercise	PL, molding, PDMS	2.3–6.1 μL	57
3D-printed microfluidic collection patch	Capillary bursting valves	Cu^2+^, Cl^−^, glucose, pH	Various	Exercise, sauna	Resin printed microfluidics, PDMS	ns	58
3D-printed microfluidic patch	Capillary bursting valves	Cl^−^	Forearm	Exercise	Resin printed microfluidics, PDMS	50.8 μL	59
Finger-tip sized sweat sensor	Hydrophilic filler + hydrogel	Cl^−^, pH, levodopa	Various (*e.g.* finger)	None	PL, molding, PDMS, SU8 filler, AG-GLY gel, PVA	750 nL	60
Evaporation pump with sponge	Hydration interface + evaporation	Cu^2+^, conductivity	Forearm	Integrated IP	Laser cutting, PET, biadhesive tape	27 μL	61
Evaporation pump with graphene	Hydration interface + evaporation + posts	Na^+^, Cl^−^, glucose, pH	Forearm	Integrated IP	Laser cutting, PDMS, LIG	76 × 26 mm^2^	22
Wearable copper sensor	Gradient surface tension	Cu^2+^	ns	Blue ink	Laser writing	ns	4
Janus textile for sweat collection	Gradient surface tension	Na^+^, lactate, urea	ns	Exercise	Hydrophobic coating, Janus Textile, PDMS	ns	62
Janus textile with integrated channels	Gradient surface tension	Total ionic charge	Chest	Exercise	Laser cutting, Janus textile, PET	ns	45
Hydrogels for sweat induction	Direct contact	Cl^−^, Na^+^, glucose	Forearm	Integrated IP	PET, ECB	ns	3
Finger-actuated microfluidic patch	Pressure	Cl^−^, Ca^2+^, glucose, pH	Forearm	Exercise	3D-printed mold, molding, silicone elastomer	ns	63

#### 3.1.1 Capillary action

Unaccompanied by auxiliary equipment, the sweat gland is the sole driving force behind sweat flow, exerting a positive pressure. The filling is further promoted by the negative pressure from the capillary action of microfluidic channels. Capillary effects are considerable at the microfluidic scale, and they occur in the microchannel at the liquid–air interface. The effect is dictated by the surface tension of the liquid, and the geometry and surface chemistry of the channel.^43^ The capillary pressure is described by the Young–Laplace equation. For a rectangular microchannel, the Young–Laplace equation relates the contact angle, microchannel size and capillary pressure by the following expression:^43^1
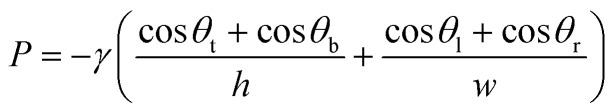
where *P* is the capillary pressure, *γ* the surface tension of the liquid in the channel, *h* and *w* are respectively the channel height and the width, and *θ*_t_, *θ*_b_, *θ*_l_ and *θ*_r_ are the top, bottom, left and right contact angles of the liquid with the channel walls.

Surface effects also have a significant role, which is why hydrophilic coatings, treatments or materials are often used to enhance capillary effects.^43^ Increasing the wettability of a surface lowers the contact angle which provides a larger capillary pressure, according to eqn (1). Material choice is crucial, as the surface free energy influences the wettability.^64^ Surface treatments offer a solution for naturally hydrophobic materials, such as PDMS, but the stability of treatments remains a challenge. For instance, PDMS will return to its hydrophobic state after conventional plasma or silane treatment within several hours.^43,65^ Other types of treatment offer greater stability like bulk modified PDMS with PEO surfactant extending stability up to 48 hours.^66^ Other alternatives to PDMS can also be used, with thiol-acrylate providing a stable, tunable hydrophilicity up to at least 14 days^67^ and the commercially available UV-curable Norland Optical Adhesive (NOA) possessing hydrophilicity lasting at least a month after O_2_ plasma treatment.^43,65^

#### 3.1.2 Microfluidic structures

To further enhance capillary filling, structures can be employed on the surface of the channel (Fig. 3Ai and ii). These maximise the contact area with the fluid thus enhancing microfluidic capabilities,^68^ such as the ‘posts’ as incorporated by Fu *et al.*^22^ or a tree/leaflike structure.^56^ Niu *et al.*^56^ adopted a bio-inspired capillary geometry to drive flow, in combination with evaporation. The patch was fabricated with polydimethylsiloxane (PDMS) and a hydrophilic agent (0.5% Triton X-100), effectively exploiting the geometry and modifying the surface chemistry to enhance capillary action.^56^

To direct flow, microvalves, such as capillary bursting valves, can be adopted.^43,47^ Here, the geometry affects the Young Laplace pressure in such a way, that flow is only facilitated through the valve after a pressure threshold is reached. This is depicted in Fig. 3Aii where the red fluid is directed through valves.^69^ Choi *et al.*^57^ and He *et al.*,^44^ also implemented capillary bursting valves to chronologically sample sweat.

Although capillary pressure facilitates filling, it does not facilitate fluid discharge after filling, which impairs long-term measurements.^70^ This is because capillary action only occurs at a three-phase contact surface (solid–liquid–gas).^22,70^ For this reason, the capillary effect can be combined with evaporation to empty the channels to enable continuous measurement, as will be discussed later. Another solution could be to add a reservoir to increase longevity.

#### 3.1.3 Hydration interface

A hydration interface can be created by placing a hydrophilic porous medium, such as filter paper, or a hydrogel at the inlet or outlet,^22,23^ as is depicted in Fig. 3B. In doing so, a pressure difference between sweat and the hydration interface is created, either by capillary porosity or osmotic pressure.^47^ It is worth noting that here, capillary action is also the driving mechanism in the porous medium. Above, capillary action was discussed in terms of the microfluidic channel network. This section focuses on techniques to improve the interface between sweat and the microfluidic channel network.

The hydrophilic porous material is typically positioned at the collection area of the patch to promote the continuous uptake of sweat. This consequently fills the microchannel with reduced sampling time and improves sampling efficiency (Fig. 3Bii).^47,60^ Instinctively, to maximise the number of accessible sweat glands and analyte volume, a large collection area is ideal for a microfluidic patch. As a downside, this also creates a large dead volume to be filled first before advancing into the channel. Nyein *et al.*^60^ tackled this problem by using a hydrophilic filler overlaying a hydrogel. This resulted in a faster filling, as the filler and gel minimised dead space and enabled quicker sweat extraction. This was earlier demonstrated by Shay *et al.*,^23^ where these effects could be attributed to the osmotic behavior of the hydrogel. A hydration interface could thus be an important addition to minimise filling time.

The downside of using hydrogels is that they become dry or contaminated after several hours of use. This requires regular replacement of the hydrogel and might limit its use to single-use sweat patch designs.^47^ In addition, they can possibly dilute the sweat composition, which is why Nyein *et al.* opted for only a thin hydrogel layer in their design.^60^ However, hydrogels also have a use for active stimulation of the sudomotor response. Sweat inducer agents, such as pilocarpine, can be added to the hydrogel and incorporated into the design of an iontophoretic patch, increasing the sweat rate.^3,47,54^ This is practical in passive cases where the wearer cannot induce sweat by active stimulation (*e.g.* doing sports).

Fu *et al.*^22^ recently developed a microfluidic sweat patch based on capillary forces and evaporation, with a hydration interface as intermediate. To increase the evaporation efficiency, a porous graphene slab was used at the end of the channel. Sweat was absorbed from the channel and driven to evaporate by the increased evaporation area of the porous graphene. This, in combination with a small pillar-like microstructure, contributed effectively to accelerating the discharging of sweat and showed robustness to deformation.^22^ A regular rinse of the porous graphene was suggested for reusability and long-term use.^22^ The employment of such microstructures is an interesting approach as it enhances sweat discharge and might be a future direction in sweat patches.

#### 3.1.4 Surface tension gradient

In order to facilitate quick filling of the microchannels, the hydrophilicity of the channel material is a key factor. Associated with hydrophilicity is surface tension. It is defined as the tension of a surface film of a liquid to minimise the surface area (Fig. 3Bi).^71^ At the microfluidic scale, the surface tension of a fluid plays a leading role and depends on the surface material and the liquid.^71^ Zhang *et al.* introduced a surface tension gradient in a smartphone-based wearable electrochemical sensor to monitor copper ions in sweat.^4^ They introduced a hydrophilic agent at the outlet to locally lower the surface tension (Fig. 3Cii). As the agent fills the channel, the surface near the outlet has the most interaction time with the agent. By the time it reaches the inlet, it will have had more time to settle at the outlet, in effect rendering the surface of the microfluidic device gradually more hydrophilic towards the outlet. This gradient acts as a passive driving force for the sweat in synergy with the capillary action, accelerating loading time. In the treatment optimisation, Zhang *et al.* described that the efficiency of the treatment depended greatly on the flow rates used to load the agent.^4^ This is an important optimisation which should be considered in any sensor using hydrophilic agents. The longevity or deterioration of the hydrophilic agent is not explicitly investigated and it can be a drawback for this type of approach.

Another demonstration of the potential of a surface gradient force is in an optical textile-based sweat sensor.^62^ Like the two-faced Roman deity, Janus textile is a fabric that contains a hydrophobic side facing the skin and a hydrophilic region on the opposite side.^45,62^ Because of this feature, the sweat is forced to the hydrophilic surface in unidirectional fashion (Fig. 3Ciii). The Janus fabric can be placed below the inlets of a PDMS layer. Optical microfibers with hydrophilic coating then extract the sweat through capillary force. The sweat production was stimulated through exercise.^62^ Liu *et al.* also used Janus textiles to collect sweat. Here, sweat was guided towards microchannels in between textile layers (Fig. 3Ciii)^45^ Similar to the hydrogels to promote channel filling and minimise empty space, this two-faced fabric could also serve the same goal, rapidly directing sweat towards the channels, by their steep surface gradient instead of osmosis or capillary action. However, the preparation of the textiles can pose more manufacturing requirements and steps, such as employing two surface modification techniques on each side^62^ or braiding and weaving of different yarns to create the Janus fabric.^45^

#### 3.1.5 Evaporation pump

Another method of promoting flow is incorporating an evaporation pump into the design of a microfluidic patch (Fig. 3Di). By combining evaporation action with capillary action, a continuous sweat collection flow can be supported or sped up without the need for an external driving force. This is often achieved by placing micropores at the outlet of the microfluidic chip or patch. By designing an array of micropores instead of one large surface, the evaporation is enhanced massively (100× for the same area), pertaining to higher diffusion rates at the edge of the pore (Fig. 3Dii).^47,48^ However, an accumulation of electrolytes at the evaporation pump can limit the lifetime of the pump.

Nie *et al.* investigated and developed a flexible microfluidic device based on an evaporation-driven pump.^70^ The device was made from laser cut hydrophilic PET layers and contained filter paper at the inlet. Because of capillarity of the paper, water was absorbed and introduced to the linear microchannel, which then met the hexagonal pore array in the final cavity. As this work only served as a proof-of-principle, no sensors were incorporated, and the flow rate was determined with Particle Tracking Velocimetry. The performance of the pump showed good agreement with the calculated performance according to evaporation theory. By changing the number of pores and the diameter, the evaporation rate can be controlled. They achieved a maximum flow rate of 0.145 μL min^−1^ (*T*: 20 °C, RH: 40%) with 61 pores (diameter = 250 μm, pitch = 500 μm). However, they did not address the potential effect of electrolyte accumulation and other biomolecules at the pores. This could substantially influence the evaporation process and block the pores over a longer time span. This may be an issue for long term sweat analysis as it can also affect the diffusion of highly concentrated interferents to the sensing area.^70^

Chen *et al.* incorporated an electrochemical sensor in an evaporation-driven microfluidic device.^46^ Instead of PET, this device is fabricated using three layers of PMMA. Here, there are three outlets in the form of micropore arrays, containing 37 pores each (Fig. 3Di). The inlet is open to air, so backflow could potentially occur if the evaporation at the inlet exceeds that of the outlets. However, this inlet was closed with PDMS after introducing a sample with a syringe pump and the device under wearable conditions would likely not have an open inlet.^46^ This design is yet to be tested under wearable conditions. It would be interesting to see a biosensor incorporated with an evaporation pump and to investigate the effects of prolonged evaporation on the reliability and flow of the sweat sensing device. Moreover, examining the on-skin performance of these sensors is also the next step. It can be expected that evaporation rates increase when the sensor is attached to the skin due to the temperature rise and fluctuations during sports practice.

Niu *et al.* recently developed a wearable electrochemical sweat sensor inspired by tree transpiration. They designed a leaf-like outlet adopting evaporation and secretion through ‘air holes’ to improve performance. The sweat production rate is increased by a microheater (45 to 47 °C), whilst also subjecting volunteers to exercise. It is reported to achieve a 16 μL min^−1^ sweat collection efficiency.^56^ However, no further elaboration on the outlet design and its effects on the flow rate in the patch is provided.

Fu *et al.* designed an evaporation pump using a porous medium.^22^ On one hand, the porous graphene creates a hydration interface with the channel, as discussed previously. On the other hand, the porous graphene creates a (micro)pore interface with the air when the channel is filled, significantly increasing the contact area of sweat with air due to its porosity. To test for reliability, 5 μL droplets were added to the evaporation series. The evaporation rate remained fairly constant at a rate of around 0.4 μL min^−1^ mm^−2^. This was sufficient to measure physically active participants during 180 min. For long continuous use, a regular rinse of the evaporation pump with a small amount of water is suggested.^22^

### 3.2. Fabrication techniques for wearable microfluidic patches

In this section, materials and fabrication techniques for wearable microfluidic sweat sensors are discussed. Effectively, this supplements the reader with knowledge of the limitations and benefits of certain materials, which are typically used for wearable microfluidic applications. Microfluidic wearable sweat sensors are in contact with the skin and are therefore distinctly different from traditional microfluidics. Not only does the patch have to be biocompatible, but it must also be comfortable to the user and provide good mechanical stability.^2^ For this reason, materials with a low Young's modulus are often selected to match the skin's elastic properties. Fabric, paper and flexible polymers meet these requirements.^2,62,72^

Fabric has good absorbing capabilities and is attractive for its everyday appearance in clothing. The fabric is usually made from synthetic or natural fibres coated with a conductive polymer or other modification which renders it sensitive to a target biomarker in sweat.^10^ Washable textile-incorporated sensors are an active field of research for non-invasive healthcare monitoring,^45,73,74^ but out of the scope of this review. It is worth mentioning, however, that some modified textiles are used for unidirectional collection of sweat in combination with polymer microfluidics, monitoring ionic compounds and sweat rate.^45,62^

A 3D printed composite silk film has also been developed for microfluidic wearable sensing using sacrificial molding. The authors were capable of printing complex three-dimensional shapes with good tensile properties, biocompatibility and self-healing ability.^75^

Apart from silk, paper has also been used for the fabrication of microfluidic channels. Paper is low cost, easy to use, foldable and most importantly, has good capillary effect for fluid flow and the collection of sweat.^72^ A smartwatch integrated paper-based microfluidic patch was developed, where paper was folded to create a three-dimensional microfluidic structure. The paper was treated with wax to form a hydrophobic barrier. While the paper patch was convenient in wearing and implementation, it remained a challenge to direct the flow from the inlet to the outlet and provide a fresh flow for reliable analysis. Nevertheless, paper could provide a sustainable, point-of-care solution in the future.^72^

However, the most frequently used materials for microfluidic sweat sensors are the polymers polydimethylsiloxane (PDMS) and polyethylene terephthalate (PET), as can be seen in Table 2, although polymethyl methacrylate (PMMA) is also commonly used for microfluidics. Aside from biocompatibility, they are low cost and provide electrical insulation.^2^ PDMS and PMMA can also be modified to exhibit more hydrophilic properties by gas phase treatments with plasma, ozone or UV light or modifying the surface with hydrophilic coatings.^43^ This is a desirable property for microfluidic sweat sensors, as discussed in the previous section. Polyimides (PI), polyurethane (PU), and polyvinyl alcohol have also been used.^2,9,75^ Moreover, microfluidic systems made from PET have been integrated into textiles to improve wearability.^45^ By doing so, better breathability than a patch is achieved, preventing sweat gland occlusion, while still benefitting from microfluidics.^45^ Sweat gland occlusion leads to compensatory sweating, which is discussed in section 4.4.

**Table 2:** Overview of recently developed flow rate sensors for sweat analysis. Ns = not specified, IP = iontophoresis, SR = sweat rate

Type	Material	Test range	Configuration	Volume/size	Remarks	Validation	Ref.
Continuous impedimetric measurement	PET	ns	Patch	24.3 μL	Conductivity dependence	IP and exercise	51
Resettable discrete impedimetric measurement	PET, PDMS, SAM	0.25–4 μL min^−1^	Forearm patch	10 μL	3–5 time usage selective to flow	Syringe pump, exercise	20
Discrete impedimetric sensing	Paper	3–14 μL min^−1^	Tabletop	82 μL	Single-use selective to flow	Droplet at inlet	76
Interdigitated electrodes for resting sweat flow	PDMS, SU8 filler, AG-GLY gel	2 nL min^−1^–3 μL min^−1^	Patch	At least 750 nL	24 h measurement, selective, needs SR estimate	Syringe pump, on-body tests	60
Self-resettable impedimetric measurement	PDMS, trimethoxysilane	0.2–4.0 μL min^−1^	Patch	333 μL	Modular design selective to flow	Syringe pump, exercise	77
Microbubble impedimetric measurement	PET, 5% Pico-Surf	0.6–10 μL min^−1^	Patch	At least 7 μL	Selective, robust to environment	Syringe pump, exercise, IP	78
Continuous impedimetric measurement	PET	1.4–3.0 μL min^−1^	Forearm patch	27 μL	Conductivity/SR dependence	Injections, IP	61
Continuous capacitive measurement	PET	0.5–1.5 μL min^−1^	Patch	ns	Ionic content dependence	Syringe pump, IP, on-body tests	79
Capacitive droplet counter	Glass	21–313 nL min^−1^ cm^−2^	Patch/finger clip-on	25 × 15 mm	Variable droplet volumes, selective	IP	80
Closed droplet counting method	PET	1–2.5 L min^−1^	Patch on forehead	ns	Orientation correction reliable up at 7000 s 170 μL sweat throughput	Syringe pump, thermal stress	81
Compartment based conductance method	PET, PU-coating	12–60 μL min^−1^	Back patch	10 × 70 μL	Identified conductivity dependence	Syringe pump, exercise	55
Segmented quantitative conductivity measurements	PDMS	0.3–1.5 μL min^−1^	Patch	80 μL	No overlap in conductivity comparison	Syringe pump, on-body tests	82
Digital droplet volume dispensing method	Acrylic sheet, PET	25 nL min^−1^–900 μL min^−1^	Wristband	1 cm Ø excl. wearable	200 hours without missing an event	Syringe pump, exercise	83
Droplet counting method using impedimetric sensing	PDMS	0.1–2.0 μL min^−1^	Tabletop	1 cm Ø excl. wearable	At least 6 hours at 1 μL min^−1^ cm^−2^	Syringe pump	84
Droplet counting method using impedimetric sensing	PDMS	0.5–20 μL min^−1^ cm^−2^	Wearable band	ns	ns	Syringe pump, exercise	37
Bursting valves for chrono-sampling of sweat	PDMS	Up to 1 μL min^−1^	Patch	Up to 300 μL	Flow results within a factor of absorbent pads	Exercise, thermal stress	57
Colorimetric image analysis method	Macroduct sweat collector, dye	0.39–4.89 μL min^−1^	Wristband	ns	ns	Syringe pump, exercise	85
Colorimetric method for flow rate	Roll-to-roll thin film elastic polymer	0.4–6 μL min^−1^ cm^−2^	Patch	130 μL for sweat rate	Excellent flexibility. Max. 1.5/2 hours	Exercise	8
Environmentally degradable colorimetric	Thermoplastic copolyester	Avg 0.97 μL min^−1^ (exercise)	Patch	20 μL	ns	Exercise, thermal stress	86
Sweat rate device	Elastomer and cellulose	And 0.22 μL min^−1^ (sauna)	Patch	20 μL	ns	Exercise, thermal stress	
Resettable microfluidics for hydration (visual inspection)	PDMS	ns	Patch	25 μL	Resistant to motion	Exercise, thermal stress	87
One-dollar paper-based colorimetric chip for sweat rate	Paper, paraffin wax	998–1710 g h^−1^ m^−2^	Patch	At least 14 μL	ns	Droplet testing, exercise	88
Evaporation equilibrium	PET	40–160 nL min^−1^	Tabletop	ns	Not tested on-body pore blockage dependency on environment	Syringe pump	70
Indirect derivation from sensor properties	PET	0.2–1.2 μL min^−1^	Patch	ns	Dependence on sweat contents max 0.24 μL min^−1^	Syringe pump	46
Thermal actuator based flow method	PDMS	0–5 μL min^−1^	Patch	11.2 × 24.8 mm incl. electronics	Reliable under temperature changes, airflow over sensor, mechanical oscillations (5 Hz) and over 1 hour of testing	Syringe pump, exercise	89
Thermal actuator based flow method	PDMS	<0.15 μL min^−1^ cm^−2^	Wristband	At least 35 μL	18% error on accuracy	Syringe pump, exercise	90

An additional consideration in polymer selection for microfluidic sweat sensors is the free volume properties of these materials. The free volume represents the unoccupied space between polymer chains, allowing diffusion of small molecules, leading to water uptake and pervaporation.^58,91,92^ Polymers with higher free volume, such as PDMS, have a high permeability to water vapor.^93^ This may introduce errors in measuring sweat rate, particularly in low-sweat-rate individuals. To address this, surface treatments or coatings can be applied to reduce water pervaporation^93^ while maintaining the polymer's flexibility and biocompatibility.

Often, polymeric substrates are patterned or moulded. Hence, a few fabrication techniques will be discussed for patterning microfluidic channels. Generally, photolithography is used for rigid polymers, although it can be used with soft polymers under special treatments. It has negligible channel roughness, but has a much slower processing cycle, capable of only printing two-dimensional lateral structures and requiring a clean room facility.^71,94^ For flexible polymers such as PDMS, soft lithography can be used. It does not require sophisticated equipment, apart from a lithographically made master, which the precursor of PDMS is poured over and then cured.^95^ The mould or master can also be 3D-printed or created in another system, and this allows for rapid prototyping. With this technique micro- and nanosized structures can be achieved.^96^

A polymer sheet can be bonded to the top to close off a channel. Many polymers are bonded using temperatures above their glass phase transition temperature. Polymers with low surface energy such as PDMS can be bonded through plasma bonding.^71,97^ For three-dimensional designs and microfluidic channels specifically, sacrificial moulding is an emerging fabrication method. A sacrificial mould is encapsulated by PDMS or another material. Once this is hardened, a solvent is introduced to the mould and subsequently dissolved.^75,94^ Traditional moulds can be reused as the PDMS is simply peeled off, as opposed to sacrificial moulds.

Alternatively, channels can be laser cut in a polymeric sheet and subsequently laminated. This does not require a cleanroom and is thus suited for rapid prototyping. However, laser cutting creates more irregularities at the channel walls than lithography. For smoother channels, optimisation is required.^94,98^

**Fig. 4:**
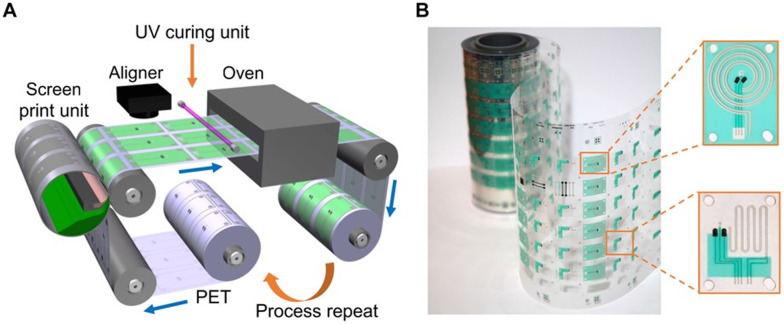
A) Illustration of the roll-to-roll (R2R) fabrication technique. B) Physical prints presenting microfluidic structures. Reprinted with permission.^51^ Copyright 2019, *Science Advances*.

Another technique that can be used in microfluidic fabrication is the roll-to-roll (R2R) technique. R2R is the most efficient method for high-throughput rapid fabrication of microfluidic channels^51^ (Fig. 4). A paper roll is coated with PDMS. A master roll with a design imprints the PDMS in continuous fashion. This is a quick way to build a larger volume of devices, but high investment is needed.^94,99^

Research on sweat sensing devices seem to primarily focus on wearable and flexible materials that can conform to the skin. If the right location on the skin is used, rigid structures may also be used for sweat sensing, as long as good contact is ensured through flexible, adhesives. One benefit of a rigid design is that rigid collection wells can avoid the collapse of the wells during the exertion of external forces. Furthermore, a flexible design might artificially force fluid in the channel upon deformations.^60^ Rigid structures can easily be achieved through 3D printing, which is efficient in printing complex compounds, whilst offering low costs and simple manufacturing.^10^ Stereolithography (SLA) 3D printers with photosensitive resin provide the best performance in resolution, capable of printing microstructures (>100 μm) and surface roughness for microfluidic features.^21,58,59,94^ Resins are typically hydrophobic, but the surface can be treated to exhibit hydrophilic properties.^100^ Alternatively, a filament 3D printer can also be used for a disposable microfluidic solution.^101,102^

For on-body sweat sensing, resin 3D printing has been adopted by printing a microfluidic skeleton.^58,59^ Yang *et al.* used acrylate based resins for spectroscopic and fluorimetric analysis, where the most hydrophilic resin yielded a contact angle of around 60°. A methylacrylate resin provided the best overall performance, along with a resolution of 200 μm.^58^ Another on-body tested sweat system adopted digital light processing (DLP) printing for colorimetric tests.^59^ The wearable 3D printed sweat systems show feasibility for 3D printing small microfluidic structures for sweat collection and analysis.^58,59^

A well-known rigid microfluidic material category is glass. Glass-based microfluidic devices are valued for their biocompatibility, chemical inertness, transparency, and hydrophilicity, commonly used in a bench-top environment.^103^ Fabrication typically involves photolithography combined with wet or dry etching techniques, allowing precise microchannel designs with smooth walls for consistent fluid behaviour and channel sizes down to tenths of nanometres.^103^ These properties enable precise sweat transport and controlled fluid flow, also in low sweat rate conditions. Despite these advantages, glass's rigidity and brittleness limit its use in flexible wearable applications.^103^ Recently however, a glass-based microfluidic sweat sensor was introduced for low flow rates, which will be discussed in section 3.3.1.^80^

The fabrication methods of all devices mentioned in this review can be found in Tables 1 and 2.

### 3.3. Current wearable sweat sensors for detecting perspiration rate

In physiological research, gravimetric methods such as absorbent patches and ventilated capsules are adopted frequently. The former method uses the collection of a certain sweat mass over time to determine flow rate, while the latter is able to continuously measure sweat rate based on humidity after actively evaporating the sweat by nitrogen gas or dry air.^49,55^ Other, wearable hygrometer-based methods exist for measuring sweat production, but this is out of the scope of this review.^104–106^

**Fig. 5:**
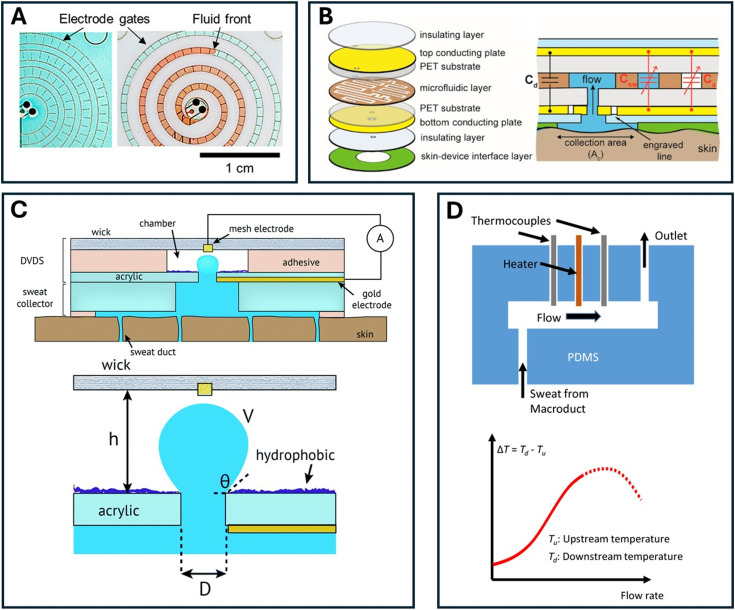
Schematic overview of the presented flow rate measurement techniques (excluding the indirect derivation from the sweat composition). A) Illustration of impedimetric measurement showing interdigitated electrodes in a spiral channel with a propagating fluid, reprinted with permission.^20^ Copyright 2022, *ACS Sens.* B) Illustration of a continuous capacitive flow rate sensor, where the microfluidic channel resides in between the conducting plates, changing the capacitance as result of different ratios of sweat (Csw) and air (Ca). Reprinted with permission.^79^ Copyright 2020, *ACS Sens.* C) A schematic overview of a volume dispensing system, illustrating the droplet before an absorption event with the wick takes place. The height (*h*), contact angle (*θ*) and diameter (*D*) of the opening influence the absorption frequency with a given flow rate. Reprinted with permission.^83^ Copyright 2018, *Lab Chip*. D) Illustration of the calorimetric method, consisting of a heater equally spaced between two thermocouples. Reprinted with permission.^90^ Copyright 2018, *Sensors*.

To measure flow rate in a wearable sweat sensor, the means are limited, as there is no room for large equipment and the use of probes can interfere with the measurement. Therefore, methods for determining real-time flow rate with the ability to be miniaturised have been developed for the integration in wearable devices. The current flow rate measuring methods are organized based on their key working principle and evaluated in this section as seen in Fig. 5. In total, these methods include: I) electrochemical methods (3.3.1), II) mechanical methods (3.3.2), III) optical methods (3.3.3), IV) indirect (bio)chemical method (3.3.4) and V) thermal methods (3.3.5).

An overview of the discussed sensors can be found in Table 2. Notably, the majority of sweat sensors have been tested during exercise.

#### 3.3.1 Electrochemical methods

Typically, impedance measurements are adopted to measure flow rate based on channel filling. Electrodes can be integrated into the channels of a microfluidic sweat patch. When the channels are filled with sweat, the electrodes are covered and the conductance will increase, which can be easily registered with an impedance measurement. In early designs, the electrodes were placed parallel to the microfluidic channel. Upon further filling, the reduced impedance could be calibrated for real-time sweat rate measurements.^51^ However, the sweat rate measurement is less accurate due to its susceptibility to changes in sweat composition,^20,55^ as it does not only measure fluid volume but also the resistivity of the fluid itself, which might be subject to change.

A way to circumvent this is by opting for an interdigitated set-up, which is also how Bariya *et al.*^20^ improved on a previous continuous design.^51^ The electrodes are now screen-printed perpendicular to the microchannel and the changes between the electrode pairs are monitored (Fig. 5A). This results in a discrete measurement: the fluid front passes electrode spokes, which results in a qualitative conductance peak, instead of a continuous signal. A higher flow rate can be identified in the form of a higher number of checkpoint crossings within a certain time period. The flow rate is retrieved based on calibrating the crossing frequency with known flow rates.^20^ This method is the most commonly used in sweat rate sensor patches (Table 2).^20,60,76,77,82^

Despite being relatively simple and easy to incorporate in a sweat sensor, the time of measurement is ultimately dependent on the channel length and the sweat rate. Because of inter-individual differences in sweat secretion rates, the monitoring time would differ per patient. Another concern is that of contamination of the channels over various measuring cycles. This results in measurement failure due to irregular filling of the channels.^20^ For the development of a reusable device, this problem should be tackled, for instance *via* periodic flushing of the channels with water.

A creative solution was found by Lin *et al.*, where they measured the propagation of a microbubble as it passed sensing electrodes for iontophoretically- and exercise-induced sweat profiles.^78^ The bubble is generated through electrolysis and then transported along the channel guided by the sweat flow. It provides a means of measuring flow, even when the microfluidic channel is completely filled. This method of discrete impedimetric sensing showed good agreement with the expected physiological response of the tested subjects in both conditions, measuring sweat rates from 8 μL min^−1^ down to approximately 0.7 μL.^78^

Liu *et al.* designed a patch that relies on interdigitated electrodes, but without the limit of channel size by providing an adaptively resettable microfluidic patch.^77^ As the channel fills, the sweat sequentially passes the electrodes. When the sweat reaches the outlet, it interacts with the absorbent to separate the fluid volume. In addition, the authors use a vent structure to maintain the air–liquid pressure equilibrium for better separation in the microchannel. To prevent leakage, stop valves are employed. These are hydrophobically treated and have a geometry with a bursting pressure that surpasses that of the microchannel. Therefore, the sweat will proceed along the main microchannel without leakage. Moreover, the design is reusable. The absorbent wick can easily be replaced. This design could process over 300 μL of sweat in a single measurement, outperforming other sensors.^77^ Interestingly, it is compatible with different absorbents, and it is claimed to remain stable under various body movements and interference.^77^

Yang *et al.* pursued a continuous monitoring concept for copper detection. Here, they measured the flow rate in order to normalise the copper concentration, measured by electrochemical detection.^61^ To omit channel contaminations, a sponge was used at the outlet to wick the fluid as it evaporated on the air interface. They chose a parallel sensor for measuring fluid admittance, which can be sensitive to a change in the ionic composition of sweat, as mentioned previously.^20,82^ It is important to consider that the continuous impedimetric flow rate sensor, which has an error associated with ionic sweat components,^20^ is used to normalise the copper concentration from the electrochemical copper sensor, which itself is stated to have an error created by sweat rate.^61^ The discrete spoke method for measuring flow rate would increase the accuracy of measurements by relying on electrode crossing events and not on the measured conductivity. Further research should be encouraged to take the biosensors' response to flow rate into account.^107^ Komkova *et al.*^107,108^ measured the effects of flow on commonly used Prussian blue based (bio)sensors and they were able to explain these effects for analyte influx and reaction product outflux through transport limitations. In spite of the drawbacks of impedance measurements, such as the dependence on channel length, the impedimetric method is appealing for its relative simplicity and good reproducibility, even at lower flow rates down to 2 nL min^−1^.^60^ Nevertheless, the evaluation of the specificity and long-term use of the electrodes should be taken to ensure continuous impedance measurements.

A continuous capacitive method for flow rate measurement can also be used (Fig. 5B).^79^ This technique uses parallel capacitor plates above and below the layer containing the microfluidic channels, suitable for layer-on-layer fabrication.^79^ The capacitor presents a linear relationship between the capacitance change and flow rate as more sweat fills the channels between the plates. The results show similarity to the commonly used Macroduct collection device.^79^ However, similar to continuous impedimetric measurements, it is expected that a change in ionic contents during measurement also contributes to the measured capacitance.^109^ With more complex manufacturing and geometries, real time capacitive flow measurements can be very precise with the ability to measure sub-nanoliter changes for microfluidic applications.^68^ However, for multiple measurements, this method is susceptible to interference from sweat residue in the microchannels, compromising the technique.^20^ The sensitivity to variable sweat composition remains to be examined.

Moonen *et al.* developed a capacitive sweat rate sensor intended for analysing sweat at rest and capable of measuring low flow rates by counting droplets.^80^ The glass-based microfluidic device operates by separating droplets at the inlet and transporting them using electrowetting-on-dielectrics. Here, the wettability of a liquid increases when a voltage is applied, which enables precise movement control of the liquid.^80^ One of the dielectrics is used to sense the passage of a droplet through a capacitance change as the air is replaced with water. A constant volume is assumed to estimate the flow rate.^80^ On-body tests after iontophoresis showed an average flow rate of 3.9 nL min^−1^ and this was converted to a sweat rate of 81 nL min^−1^ cm^−2^, which is within the resting sweat rate range. The authors intend to continue with the concept and investigate its feasibility for clinical use.^80^

Electrochemical methods are a simple, robust means of measuring flow. Continuous impedimetric and capacitive methods can be adopted, but an interdigitated electrode design is most effective in selectively measuring flow. A downside is that these methods monitor a fluid front. Once the total channel capacity is reached, the measurement ends. To prolong measurement time, the channel can be emptied. However, reusability is challenging due to salt deposition in the channels, although through wicking the channel can be reset, as recently demonstrated.^77^

#### 3.3.2 Mechanical methods

Another recently developed method for establishing flow rate measurements is digital volume dispensing systems (DVDS), as introduced by Francis *et al.*^37,83,84^ It is a discrete method that relies on the temporary shorting of periodic droplets generated between two electrodes with a small, constant voltage applied (0.5 V). Sweat passes the first electrode as it enters the droplet chamber. The bottom of this chamber contains the inlet hole and a superhydrophobic surface to promote a consistent spherical shape of the droplet (Fig. 5C). A second electrode is placed on the top part of the chamber. The top part consists of an absorbent material which wicks the droplet as soon as it comes in contact with the absorbent. The droplets are assumed to be spherical and thus from each droplet breaking event, the flow rate can be extracted from the volume of the droplet over time. The height of the chamber mainly controls the volume of the droplet. The droplet has a rather small volume of 10–100s of nL and a high surface tension. The authors found the Bond number sufficient for minimal movement, which means that the surface tension is dominant over gravity. However, they also suggested limiting the chamber height to 1600 μm to account for 10*g* acceleration. This is to keep the surface tension dominant even under body movements during on body testing.^83^ This system shows great durability and reliability: it can continuously operate for up to around 200 hours at 0.1 nL min^−1^ (syringe pump) without missing an event. The authors validated the DVDS with on-body testing, comparing it to gravimetric measurements. Despite showing higher sweat rates then the gravimetric method, the DVDS worked as intended according to the pre- and post-calibration of the device.

The authors identified three challenges with the DVDS. One was air bubble formation, causing unexpected results at the start of use. Another major challenge was that the wick could dry out for smaller flow rates which causes a longer breaking time and changes in droplet volume. This could be solved by more sophisticated construction methods or an optimised electrode type. The used mesh electrode was noted to interact differently with the droplet under wet and dry conditions. Finally, salt accumulation in the wick and eventually in the chamber governs the life span of the device. A balance must be found in fluid retention as to keep the electrode wet, and fluid dissipation to minimise salt accumulation and ultimate clogging.^83^

A similar, yet different approach was taken by Wang *et al.*^37^ They incorporated a vertical fluid rate sensor in a wearable platform which also detected electrolyte concentration. While this method also relies on a wicking material to break surface tension and absorb droplets, the droplet is allowed to fill the chamber completely until it touches the absorption layer. This creates a relatively constant volume to be absorbed. An electrode is placed at either side of the chamber, measuring conductivity. The diameter of the chamber and the distance of the electrodes to the absorption layer were optimised to provide a maximum conductance signal.^37^ They mention that lower filling rates remain a challenge due to the longer filling times, because this provides less “real-time” data and potentially bias. Aside from this, fabrication is simple, and similar flow rates during testing were measured compared with the absorbent pad. The droplet sensor shows fluctuations in flow rate, but this could be attributed to the natural pulsating mechanism of sweat secretion.^37^ Volume dispensing systems relying on the discrete absorption of small droplets are attractive for their life span, compact size and selectivity driven by flow. Salt accumulation as well as saturation of the wick in the wick remains a challenge. Another challenge resides in ensuring consistent droplet volumes under motion conditions and deformation.

#### 3.3.3 Optical methods

Another distinct approach is using visual inspection to give an indication of the flow rate.^70,85^ For example, Nie *et al.*^70^ developed an evaporation based digital microflow meter that operates in the range of 30–250 nL min^−1^. The outlet consists of an array of micropores. For certain flow rates, not all pores will be reached as the flow rate equals the evaporation rate before that time. The flow rate can thus be determined from the amount of visibly wetted micropores. While this requires a camera setup, it is an interesting approach which also provides a good estimation of the actual flow rate. Possible issues may arise when the pressure drop over the whole system is too large. The liquid may be forced out of the pores, or the assumption of flat liquid menisci does not hold due to deformation.^70^ For wearable applications, the feasibility of such a design should also be investigated under dynamic movements and under different device orientations.

**Fig. 6:**
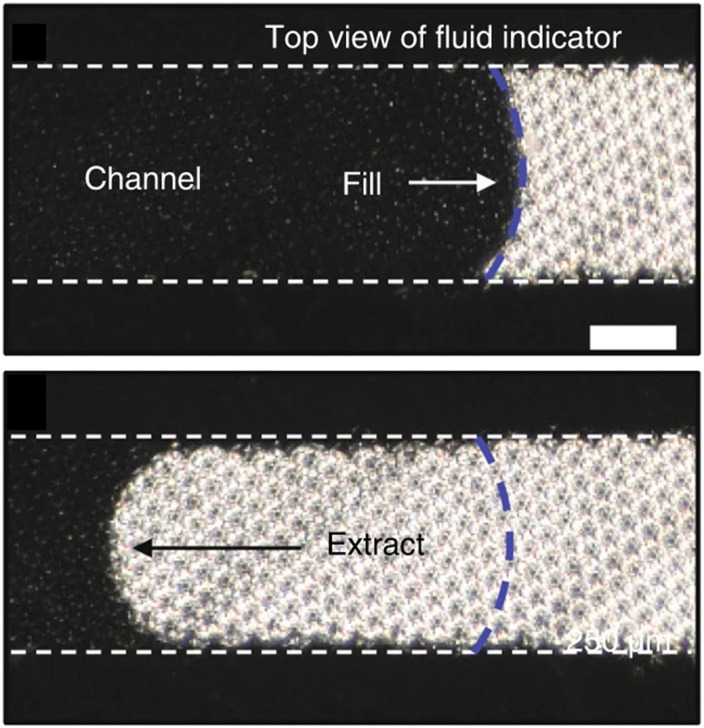
The reversible fluid indication method of Reeder *et al.* The PDMS and sweat have a similar refractive index (∼1.4, ∼1.3 respectively), limiting reflectance. When the channel is empty, the microstructures reflect 60% of the light. Reprinted with permission.^87^ Copyright 2019, *Nat. Commun.*

Matzeu *et al.*^85^ made use of the existing infrastructure of the Macroduct sweat collector to create another visual method for determining flow rate. By placing black food colouring at the entrance of the collector, they coloured the sweat. This created a contrast which could be filmed as it propagated.^85^ This colorimetric method does not rely on the electric properties of sweat, as opposed to electrochemical methods, which makes this technique attractive.^84^ In addition, it does not require the integration of a power unit, because all analysis can be done off-site by camera or mobile phone.^84,86^ A sequential filling method could also be used to determine sweat rates. With the use of capillary bursting valves and a multitude of compartments, one is able to roughly determine the flow rate.^57^ The design is more complicated than the linear filling of a channel, but can provide quick analysis. The patch design was varied in number and size of the compartments.^57^ Due to the simplicity of the colorimetric method, its use has been widespread. The Rogers Research Group has over 5 years experience with the use colorimetric sweat sensors and have recently contributed bringing a colorimetric sweat rate sensor to market.^8,110,111^ The single-use patch is intended for athletes to visualise and measure fluid loss and make personalised hydration recommendations for better recovery.^111^ Colorimetric method has also been employed to analyse sweat composition by binding biomarkers, altering the colour in a chemical process.^2^ It does, however, face some challenges. Like the interdigitated electrodes, this method is ultimately dependent on channel volume, and the dye must not interfere with other measurements. However, by adding a texture on a PDMS surface for instance, the use of dyes can even be omitted (Fig. 6).^87^ Reeder *et al.* designed a manually resettable patch for the hydration of athletes. The patch adopts the optical properties of the PDMS microstructures in such fashion that an empty and filled segment of the channel can be discerned.^87^ The optical method provides real-time information on the filling of a fluid front, merely requiring a contrast between air and sweat and a microfluidic channel. Stand-alone it is a passive wearable device, where filling can be monitored using manual inspection or external devices. The user input may not be practical during a field hockey match, for example and poses a drawback depending on the application. Other challenges reside in the limited volume capacity and dye interference.

#### 3.3.4 Indirect (bio)chemical method

As alluded to earlier, some biosensors may exhibit a flow rate or mass transport dependence.^107,108^ This feature can be exploited to extract the flow rate potentially.

One method uses the limiting current of a sensor to be related to the flow rate instead of measuring the impedance.^46^ A limiting current is the result of the sensor response to flow, where the mass transfer and not the reaction rate is the limiting factor. The simple, easily fabricated structure consists of three electrodes. These results showcased good reproducibility as similar but not identical responses were observed over multiple measurements.^46^ However, an NaCl solution was used to mimic the sweat and no real sweat samples were tested. Also, long-term stability of the device was not investigated, and the measurements were performed in a short time span (<2 min, no mention of longer measurements). Interestingly, the equation used to relate volume flow rate to the limiting current also contains a variable for the ionic composition of sweat. Thus, this approach needs to be further investigated to account for potential variability in the sweat composition during the test.

Another indirect method can be to exploit the ionic content for sweat rate measurements. Specifically, sweat conductivity could serve as an indicator of sweat rate.^55^ Sweat conductivity has been shown to be directly related to sweat rate. The linear relationship can be used to accurately monitor sweat changes during exercise.^55^

Similarly, Komkova *et al.* have investigated the feasibility of a wearable device for simultaneous monitoring of sweat rate and lactate content.^107^ Sweat lactate is thought to relate to local sweat gland recruitment.^30^ Komkova *et al.*^107^ identify that lactate measurements in flow are prone to errors since the lactate sensor is used in a hydrodynamic environment. Without a flow-independent sensor, the same arguments hold for the in-flow monitoring of other analytes. Thus, the fluctuations that can lead to an analytical error can be used to indirectly distil the flow rate. Essentially, the flow rate-lactate concentration relation for some sensors could be used to indirectly extract the flow rate. In this case, a Prussian blue electrode with siloxane membranes and immobilised lactate oxidase was suggested for flow-susceptible lactate measurements. For measurements insensitive to flow input within the lactate ranges (up to 80 mM), the same sensor is used, but now with a composite membrane of perfluorosulfonated ionomer (PSFI)-siloxane, in which sensitivity is slightly traded for selectivity.^107^ This type of flow rate method is easy-to-use, but it can be difficult to fabricate depending on the type of electrode. The electrodes retain over 90% of the initial sensitivity within several hours,^107^ limiting the reusability. In fact, the long-term stability of the biosensor needs to be thoroughly studied to avoid changes in performance owing to biosensor degradation and not flow rate changes. Nonetheless, this out-of-the-box technique was worth mentioning to further stimulate innovation in flow rate methods.^108^ In general, these techniques can be used, targeting the flow rate, but they are less reliable. Monitoring conductivity changes to infer the flow rate remains complex due to other factors.^8^ The potential for sweat rate measurements based on a flow-dependent and flow-independent sensor remains to be investigated further.

#### 3.3.5 Thermal methods

The final method to discuss in this review is the calorimetric method,^89,90,112^ where heat is added to the system to detect flow (Fig. 5D). Kwon *et al.*^89^ achieved this in the form of a small thermal actuator, accompanied by a thermoresistors up- and downstream to the heater. It is able to continuously measure the flow rate through the temperature difference between the thermoresistors in a short linear channel.^89^ If no flow is present, the heat would be dissipated in a symmetrical manner. If the flow is present, a temperature difference will be measured between the up- and downstream thermistor. This method was extensively tested for reliability under various conditions such as temperature changes, airflow, mechanical oscillations (5 Hz, 2 mm amplitude), and 1 hour of testing. By using a separate reference thermistor, any environmental effects on the thermistors could be corrected in this way. This method enabled flow rates from 0–5 μL min^−1^ to be measured.^89^ As the flow rate reaches a critical maximum, the cooling effect of fluid flow exceeds the heating effect.^18,90^

Similar to the DVDS, the calorimetric method does not rely on channel length or the electric properties of sweat to provide a signal. Besides, it requires integration of a power supply and heater. Measuring flow rate by temperature difference and sweat composition *via* colorimetry can be done simultaneously. This has shown good results in terms of precision and reliability. However, when used in conjunction with other biochemical sensors, the influence of temperature variation at the site of these biochemical sensors must be considered for accurate analysis.

## 4 Key findings and prospects

This review has covered the main filling principles for wearable microfluidics, fabrication techniques and a broad spectrum of microfluidic sweat rate sensors. Sweat rate sensors are essential for sweat analysis. The sweat rate influences the sweat composition, and sweat rate varies inter-individually and depending on physical activity. By accounting for the sweat rate, the sweat composition can be standardised and normalised. Regardless of normalising sweat, stand-alone sweat rate analysis already has value in estimating hydration status and fluid loss. In this section, the key findings and prospects of microfluidic sweat rate sensing will be discussed.

### 4.1. Flow rate sensing principles

Five distinct modalities have been discussed to assess sweat rate, covering the electrochemical, thermal, mechanical and optical domains. Continuous and discrete capacitive and impedimetric methods measure fluid propagation and are the simplest to apply, but are both limited by the volume capacity of the microfluidic channel. For measurements with fluctuations in sweat rate, continuous capacitive and impedimetric measurements could be sensitive to the fluctuations of ionic content.^20^ Discrete impedimetric methods are more selective to the flow rate and the most widely used in patch designs (Table 2). To deal with the dependency on channel volume, one can tailor the channel shape to the expected sweating rate^60^ or create a resettable design as recently demonstrated for both the colorimetric/visual analysis method^87^ and interdigitated electrodes.^77^ The latter was truly continuous, because it was self-adaptable due to the wick at the end of the channel, creating a new attractive design of a widely adopted technique for continuous monitoring.

Self-resettability is also implemented in the DVDS^77^ that use a wick and a pair of electrodes to detect the periodic absorption of a droplet and subsequently deduct the flow rate. Digital volume dispensing systems thus do not rely on channel length and are capable of long-term, real-time measurements, but have more complex fabrication requirements. The DVDS are compact and generate droplet wicking events from which the flow rate is deducted. The calorimetric method provides the same benefits as the DVDS. It only needs a linear channel to continuously measure flow, but efficient power integration is required.^18^ It measures flow rate based on the temperature difference between two thermopiles, equally spaced from a heater. In doing so, the calorimetric method has shown robustness under various conditions such as mechanical vibrations and temperature changes.^89^ This type of testing is recommended for any wearable sweat sensor, in order to explicitly investigate reliability under wearable conditions, such as abrupt motions or mechanical loading.

### 4.2. Integration in multi-sensing platforms

A multi-sensing platform is most likely needed for the implementation of sweat sensors in health monitoring. A multi-sensing patch would not just contain a sweat rate sensor, but other sweat sensors for measuring sweat composition as well. For stand-alone flow rate sensor concepts, integration is the next step. However, compatibility assessment of the used techniques is needed due to the limited space and the measurement ideally taking place in one microfluidic channel. This is because the flow rate measurement techniques may influence the sweat composition, temperature and flow rate, also affecting other sensors in the integrated patch.

A colorimetric flow rate sensor for example could influence the sweat composition sensors,^8^ because the colorimetric agent could act as an interferent. Despite the absence of chemicals, the calorimetric method may also alter the measurement by heating the collected sweat. Although the temperature rise is in the order of a few degrees, it is nevertheless worth examining the implications of this for the compatibility with integrated (bio)chemical sensors. For instance, more distance between the flow rate sensor and (bio)chemical sensor might be needed to allow the sample to cool off.

### 4.3. Towards a wearable product solution

When moving towards a wearable solution for measuring sweat rate, new user-centred requirements or constraints may be considered, such as resistance to motion, or ease-of-use. Some techniques may be preferred over others to tailor the sensor for fitness, point-of-care or other medical applications. For example, interdigitated electrodes or a dye at the inlet of the channel are the simplest, reliable methods for measuring flow, excellent for short-term measurements during a sports event. However, the use of a dye alters the use of the wearable, because it forces the user to periodically watch the channel filling or use an external reader such as the camera of a smartphone.

A volume dispensing method is suggested for the most compact and long-term monitoring solution, albeit with additional tests ascertaining its sensitivity to perturbations and deformation. Besides, the calorimetric method seems to exhibit the most potential for reliable, real-time, continuous and reusable flow rate measurements, simply requiring a linear channel.^89^

Other aspects worth developing, which apply to any of the sensors, are the use of biodegradable materials^86^ and the development of a reusable infrastructure for sweat sensing devices. Maintenance of these devices may encompass periodic flushing of the channels and the replacement of a wick.^77^ This is an interesting prospect for point-of-care applications and a sustainability viewpoint.

Regarding commercialisation success, a colorimetric sensor has recently been launched for sports dehydration monitoring,^110,111^ and patents are developed for sweat rate sensors.^113^ Although more research is needed to understand the physiology of sweating and the excretion of analytes such as pharmaceuticals in sweat, it is expected that commercial medical product solutions will arise. These developments are worth keeping an eye on.

### 4.4. Accelerating research on (patho)physiological significance of sweat

Improving the understanding of sweat and its relation to diseases, drugs and health, can help improve the implementation of sweat rate sensors in healthcare. Although iontophoresis was considered and implemented in a few cases (Tables 1 and 2) the focus in the development and testing of sweat rate sensors needs to shift on a larger scale from exercise and sport towards patient monitoring.

At the same time, sweat rate sensors can be adopted as a convenient tool for (patho)physiological research. However, it is important to remember that the measurable flow rate and the physiological sweat rate it conveys are different. The volume flow in the microfluidic channel is the product of the collective rate of the sweat glands covered by the collection area of the patch. To estimate the exact sweat rate per gland, it must be retrieved by dividing the flow rate by the sweat gland density. For further accuracy, a statistical approach can be used for the sweat gland dispersion.^80^

Another effect to account for as well is that of compensatory sweating when using a collection patch. Due to sweat gland occlusion at the sides of the collection area, the sweat rate will increase locally, affecting further analysis.^45,87,114^ To increase accuracy, the sweat collector can be designed to allow for a release of sweat adjacent to the collection area to the periphery, preventing compensatory effects.^87^

If these factors are well studied and accounted for in the sensor solutions, sweat rate sensing can be a powerful tool in accelerating reliable sweat research. Harshman *et al.* found that sweat rate normalisation can correct for approximately ¾ of the error in ionic contents and metabolomic analysis. Nevertheless, this should be tested over a larger diverse population and under different conditions such as resting sweat, iontophoresis and exercise. Baker and Wolfe (2020)^13^ also advocate for further research on factors influencing sweat composition. Not only sweat flow rate, but also skin surface contaminations, byproducts of sweat gland metabolism, and secretory/reabsorptive mechanisms are influencing sweat composition. Before using sweat as an alternative for blood analysis, there must be a reliable way of investigating sweat constituents. This can start with standardising measurements for the sweat rate analysis over different body parts. The incorporation of reliable sweat rate sensors is a vital part of this. Physiological research together with a reliable measuring platform is the first step for meaningful sweat analysis that is worth your sweat.

## Data availability

No primary research results, software or code have been included and no new data were generated or analysed as part of this review.

## Author contributions

R. F. R. Ursem: writing – original draft, writing – review & editing, investigation, conceptualization, visualisation. A. Steijlen: writing – review & editing, conceptualization, visualization. M. Parrilla: writing – review & editing, conceptualization, visualization. J. Bastemeijer: writing – review & editing. A. Bossche: writing – review & editing. K. De Wael: writing – review, editing & funding acquisition.

## Conflicts of interest

There are no conflicts to declare.
